# The Absorption, Distribution, Metabolism, and Excretion of Binimetinib Following a Single Oral Dose of [ ^14^C]Binimetinib 45 mg in Healthy Male Participants

**DOI:** 10.1002/prp2.70061

**Published:** 2025-01-30

**Authors:** Dustin Huynh, Erik Hahn, Micaela B. Reddy, Renae Chavira, Lance Wollenberg

**Affiliations:** ^1^ Clinical Pharmacology, Oncology Pfizer Inc. La Jolla California USA; ^2^ Clinical Pharmacology, Oncology Pfizer Inc. Boulder Colorado USA; ^3^ Clinical Development Pfizer Inc. New York New York USA

**Keywords:** ADME, binimetinib, kinase inhibitor, MEK162, metabolism, pharmacokinetics

## Abstract

Binimetinib is a MEK1/2 inhibitor particularly active in cells harboring activating mutations in the MAP kinase pathway, especially in *BRAF* and *NRAS*. Binimetinib, in combination with encorafenib, has received marketing approval in several jurisdictions for the treatment of patients with *BRAF* V600E or V600K mutant melanoma. The absorption, distribution, metabolism, and excretion of binimetinib were evaluated by administering a carbon 14–labeled binimetinib 45 mg dose (containing 40 μCi of radiolabeled material) to 6 healthy male participants. A total of 62.3% of the radioactivity was eliminated in the feces, while 31.4% was eliminated in the urine. The overall recovery of radioactivity in the excreta for all 6 participants was 93.6% (3.27%), indicating that good mass balance was achieved. The total percentage of the dose in the excreta of all metabolites containing the N‐demethylation clearance of binimetinib by CYP1A2 and CYP2C19 was approximately 17.8%. The contribution of direct glucuronidation to the clearance of binimetinib was estimated to be 61.2% and represented the majority of the clearance. Additionally, excretion of unchanged binimetinib into the urine was estimated to have contributed 6.9% to the overall clearance. Based on study results, binimetinib is at least ≈ 50% absorbed, but based on its PK properties and because its glucuronide conjugates are unstable in the GI tract, absorption is thought to be significantly higher.

## Graphical abstract

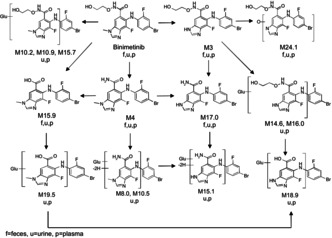

**Abbreviations**
%RA: percent radioactivity
[^14^C]: carbon 14 labeled
ADME: absorption, distribution, metabolism, and excretion
AE: adverse event
AUC: area under the curve
AUC_inf_
: AUC from time zero to infinity
AUC_last_
: AUC from time zero to the last measurable concentration sampling time
BRAF: B‐Raf proto‐oncogene serine/threonine kinase
CL/F: apparent clearance from the plasma
*C*_max_
: maximum (peak) observed plasma drug concentration
CYP: cytochrome P450
DPM: disintegrations per minute
ECG: electrocardiogram
GI: gastrointestinal
KRAS: Kirsten rat sarcoma viral oncogene homolog
LC: liquid chromatography
LSC: liquid scintillation counting
MEK: serine/threonine‐protein kinase MEK
MS: mass spectrometry
NRAS: neuroblastoma rat sarcoma viral oncogene homolog
PK: pharmacokinetics
*T*_1/2_
: terminal plasma elimination half‐life
*T*_max_
: time to reach maximum (peak) drug plasma concentration
UGT1A1: UDP‐glucuronosyltransferase 1 enzyme

## 1 Introduction

Binimetinib is an orally bioavailable, selective, and potent inhibitor of serine/threonine‐protein kinases MEK1 and MEK2. Binimetinib has demonstrated robust and selective growth inhibitory activity in a variety of cancer cell lines [1, 2, 3]. Binimetinib has shown particular anti‐proliferative activity in cells harboring activating mutations in the mitogen‐activated protein kinase pathway (e.g., B‐Raf proto‐oncogene serine/threonine kinase, *BRAF*, neuroblastoma rat sarcoma viral oncogene homolog, *NRAS*, and Kirsten rat sarcoma viral oncogene homolog, *KRAS*), especially in *BRAF* and *NRAS* (data on file). Binimetinib 45 mg orally twice daily, in combination with encorafenib (also known as LGX818), has received marketing approval in several jurisdictions for the treatment of patients with *BRAF* V600E or V600K mutant melanoma. In addition, binimetinib in combination with encorafenib and cetuximab is approved in Japan for the treatment of unresectable advanced or recurrent colorectal cancer with *BRAF* V600E mutations progressing after chemotherapy. Binimetinib is currently also being investigated in combination with multiple antineoplastic agents (chemotherapy, endocrine therapy, and/or targeted agents) in patients with selected advanced or metastatic solid tumors, such as non‐small cell lung cancer, melanoma, biliary, breast, and ovarian cancers [4, 5, 6, 7, 8, 9, 10].

Prior to this study, information on the absorption of binimetinib was available via in vitro assessment of solubility, permeability in cell‐culture monolayers and limited clinical data [1]. Though an efflux transporter substrate (for p‐glycoprotein, P‐gp, and breast cancer resistance protein, BCRP), binimetinib demonstrated high permeability in human epithelial cell line Caco‐2 monolayer assays (data on file). The dose of binimetinib (45 mg) combined with its higher solubility (> 1 mg/mL) in the acidic conditions found in the gut suggest that a dose may be fully in solution in the stomach when administered with 250 mL of water, driving pH‐shifted intestinal supersaturation of the dissolved drug that leads to improved drug absorption [11, 12, 13, 14, 15]. In clinical studies, a *T*_max_ of 2 h has been observed prior to steady state [1]. These properties suggest that binimetinib is likely fully absorbed at its current clinical dose.

The pharmacokinetics (PK) of binimetinib as both monotherapy and in combination with encorafenib has been extensively described, with plasma concentrations estimated using traditional bioanalytical methods incorporating mass spectrometric detection and non‐compartmental analyses [1, 2]. Preliminary in vitro evaluation indicated that binimetinib is metabolized by multiple routes but primarily by glucuronidation pathways (mainly via UDP‐glucuronosyltransferase 1 enzyme [UGT1A1], −1A3, 1A9, and 2B7) and to a lesser extent by oxidation pathways (mainly via cytochrome P450 [CYP] 1A2 and 2C19). The PK of the active, N‐demethylated metabolite of binimetinib, formed primarily through CYP1A2‐ and 2C19‐mediated metabolism, with each enzyme contributing roughly equally to its formation, has been previously characterized in clinical studies. The N‐demethylated metabolite (also referred to as M3, AR00426032 [N‐demethylated binimetinib]) is approximately equipotent to binimetinib but circulates at < 23% of the exposure of binimetinib across dose levels in the escalation [1]. However, the amount of this and other metabolites circulating as a fraction of total drug‐related material was still unknown. Robust knowledge regarding routes of excretion for binimetinib would allow for the identification of special patient populations that may experience higher‐than‐anticipated drug exposures (e.g., patients with hepatic and renal impairment).

The primary objectives of this study were to investigate the absorption, distribution, metabolism and excretion (ADME) of binimetinib, to assess the rates and routes of excretion of the parent drug and its metabolites and to evaluate the mass balance of total drug‐related radioactivity in urine and feces following administration of a single 45‐mg (40‐μCi) dose of [^14^C]binimetinib to healthy participants. The safety of administering this single [^14^C]binimetinib dose was also evaluated. Additionally, the PK of total radioactivity in blood and plasma and of binimetinib and its potent N‐demethylated metabolite AR00426032 in plasma was assessed. Profiling of metabolites in excreta (urine and feces) to determine key biotransformation routes and clearance mechanisms was also conducted.

## 2 Methods

### 2.1 Clinical Study Design and Treatment

**FIGURE 1:**
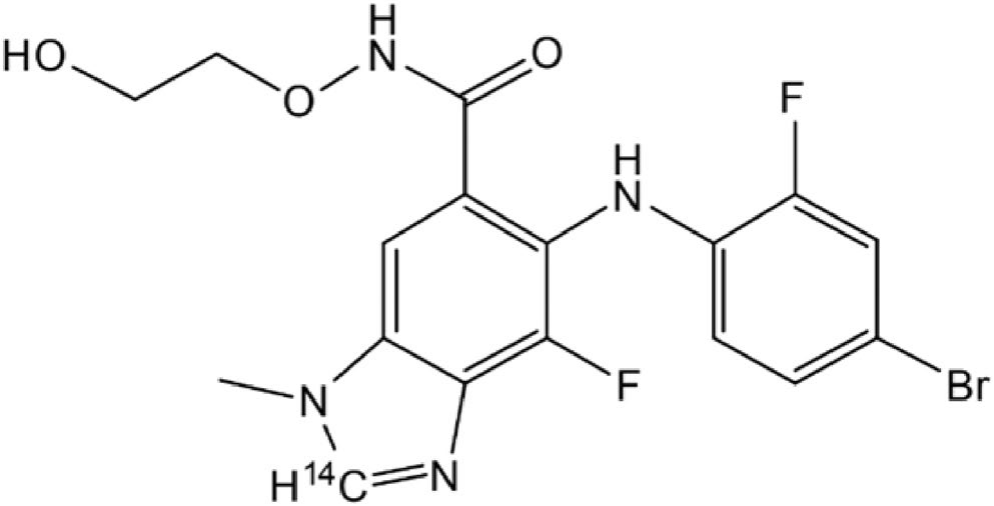
Chemical structure of binimetinib with label placement.

This study was a single‐center (Covance Clinical Research Unit, WI, USA), open‐label study to investigate the ADME of binimetinib after a single oral dose of [^14^C]binimetinib 45 mg (40 μCi) was administered to healthy male participants. The chemical structure of binimetinib and the location of the radioactive [^14^C] label on the molecule are shown in Figure 1.

Six participants were enrolled and received a single dose of [^14^C]binimetinib. This study consisted of a screening period, baseline evaluations, and a single dose administration followed by a 312‐h post‐dose observation period. A safety follow‐up assessment was conducted by phone 30 days after administration of [^14^C]binimetinib 45 mg. Participants were discharged from the clinical site 14 days after dosing, provided that the participant's recovery of [^14^C]binimetinib was ≥ 85% of the administered dose and 2 consecutive 24‐h urine and fecal collections yielded < 1% of dose. The study period was prolonged for 1 participant who had urine and feces collected on days 15 and 16 until satisfactory radioactivity recovery was confirmed.

This clinical study was designed, implemented, and reported in accordance with the International Conference on Harmonization Harmonized Tripartite Guidelines for Good Clinical Practice, applicable local regulations (including European Directive 2001/20/EC and US Code of Federal Regulations Title 21), and the ethical principles of the Declaration of Helsinki. The protocol and informed consent form were reviewed and approved by an institutional review board before the study start. Informed consent was obtained from each participant before conducting any study‐specific procedures.

Given that this was a phase 1 trial initiated before January 2017, the study was not required to be registered with ClinicalTrials.gov and a clinical trial registration number is not available.

#### 2.1.1 Dosimetry

The planned oral dose of binimetinib 45 mg contained 40 μCi (1.5 MBq) of [^14^C]. The expected radiation exposure of a participant was estimated prognostically according to the guidelines of the International Commission of Radiological Protection. This estimate was based on available human PK data, rat and monkey ADME data, and rat tissue distribution data for [^14^C]binimetinib.

The human organ doses were estimated, based on conservative assumptions, by extrapolation of systemic exposure from animals to human. The majority of the effective dose was estimated to be taken up in the colon, followed by the stomach at much lower levels. Other organs were expected to be exposed to marginal doses only. The whole‐body committed effective dose was estimated, based on conservative assumptions, to be 0.36 mSv; but the actual effective dose could have been lower. The expected radiation exposure of a participant was estimated prognostically according to the guidelines of the International Commission of Radiological Protection.

#### 2.1.2 Dose Preparation

The parent batch of radiolabeled drug substance (high specific radioactivity) was prepared by the Isotope Laboratory (Novartis, Basel, Switzerland). This batch was adjusted for final specific radioactivity by dilution with non‐radiolabeled drug substance, using a non‐radiolabeled good manufacturing practice batch of binimetinib released for human use according to predefined specifications by Novartis Technical Research and Development Quality Assurance. Each participant, therefore, received a single oral dose of [^14^C]binimetinib 45 mg formulated as a capsule (total dose, 40 μCi [1.5 MBq]) per protocol.

#### 2.1.3 Dose Administration

One capsule of [^14^C]binimetinib 45 mg was administered early in the morning to each of 6 healthy, male participants after completion of an overnight fast (≥ 8 h), and the participants continued to fast until 4 h post‐dose. Each participant was provided with 8 oz. (240 mL) of non‐carbonated water to swallow the capsule.

#### 2.1.4 Collection of Biological Samples

Urine and feces samples were collected pre‐dose, then over 4‐h intervals for the first 12 h, a 12‐h interval between 12 and 24 h, and 24‐h intervals between 24 and 456 h post‐dose. Blood samples were collected pre‐dose and every 30 min up to 2 h, then at intervals increasing from 1 to 24 h across the 14‐day study period (Table S1).

#### 2.1.5 Measurement of Binimetinib and AR00426032 Concentrations in Plasma

Plasma concentrations of binimetinib and its metabolite AR00426032 (N‐demethylated binimetinib) were determined using a validated liquid chromatography‐mass spectrometry (LC–MS/MS) bioanalytical method at QPS (Newark, DE, USA) with a lower limit of quantitation of 1 ng/mL for both analytes. D4‐binimetinib and D4‐N‐demethylated binimetinib (Isotope Laboratories, East Hanover, NJ, USA) were used as internal standards for binimetinib and AR00426032, respectively. Binimetinib and AR00426032 and their internal standards were isolated from plasma samples by solid‐phase extraction and were evaporated and then re‐constituted with acetonitrile in water (20:80, *v*/*v*) before analysis. The LC system was Shimadzu LC‐10ADVP (Shimadzu Corporation, Columbia, MD, USA) using a Luna C18, 50 × 2.00‐mm column and gradient elution (mobile phase A: water/1.0 M ammonium formate [1000/5, *v*/*v*]; mobile phase B: acetonitrile/water/1.0 M ammonium formate [950/50/5, *v*/*v*/*v*]) at a 0.4‐mL/min flow rate. The mass spectrometer was an AB‐Sciex Triple Quadrupole API 4000 using Turbo Ion Spray.

#### 2.1.6 Measurement of Total Radioactivity in Plasma, Blood, Urine, and Feces

Analysis of total radioactivity in plasma, blood, feces, and urine was performed by Covance Laboratories Inc. (Madison, WI, USA). All sample combustions were performed in a Model 307 Sample Oxidizer (Packard Instrument Company, Downers Grove, IL, USA), and the resulting radio‐carbon oxygen was trapped in a mixture of Perma Fluor (Perkin Elmer, Waltham, MA, USA) and Carbo Sorb (Perkin Elmer, Waltham, MA, USA). Oxidation efficiency was evaluated on each day of sample combustion by analyzing a commercial radiolabeled standard both directly in a scintillation cocktail and by oxidation. Acceptance criteria were combustion recoveries of 95%–105% relative to the direct analysis. EcoLite(+) scintillation cocktail was used for samples analyzed directly. All samples were analyzed for radioactivity in model 2900TR liquid scintillation counters (Packard Instrument Company, Downers Grove, IL, USA) for at least 5 min or 100 000 counts. Each sample was homogenized or mixed before radioanalysis (unless the entire sample was used for analysis). All samples were analyzed in duplicate or triplicate, as applicable, if sample size allowed. If results from sample replicates (calculated as ^14^C disintegrations per minute [DPM] per gram of sample) differed by > 10% from the mean value and sample aliquots had radioactivity > 200 DPM, the sample was re‐homogenized or re‐mixed and re‐analyzed (if the sample size permitted).

Scintillation data (counts per min) were automatically corrected for counting efficiency using the external standardization technique and an instrument‐stored quench curve generated from a series of sealed quenched standards.

Blood samples were homogenized, and duplicate weighed aliquots (≈ 0.4 g) were combusted and analyzed by liquid scintillation counting (LSC). Plasma and urine samples were separately mixed, and duplicate weighed aliquots of each (≈ 0.4 g for plasma and 0.2 g for urine) were analyzed directly by LSC. Urine volumes provided by the Covance clinical research unit were entered as a weight, assuming a specific gravity of 1 g/mL. Fecal samples were analyzed individually (samples were not combined by interval), and the weight of each individual sample was recorded. A weighed amount of water (≈ 2–3 times the sample weight, such that the homogenate weight: sample weight ratio was ≈ 3 to 4) was added, and the sample was mixed and homogenized using a probe‐type homogenizer. Triplicate weighed aliquots (≈ 0.2 g) were combusted and analyzed directly by LSC. Residual radioactivity recovered from each treatment vial was subtracted from the dose administered to the participant.

#### 2.1.7 Sample Preparation, Determination of Metabolite Profiles, and Identification of Metabolites

Plasma area under the curve (AUC) pools were prepared using the Hamilton method over 0–24 h [16]. Pooled plasma samples for each participant were extracted three times with acetonitrile. The recoveries after extraction counted by LSC averaged approximately 93%. Fecal homogenates were pooled by combining 1% of the original sample weight from each fecal portion. The radioactivity in each pool represented > 91% of the total radioactivity recovered in feces over the entire collection period. A pooled fecal sample from each participant was extracted three times with acetonitrile. The average extraction efficiency was determined by LSC to be 92.0%. Urine samples were pooled by combining 0.2% of the original sample weight from each urine collection interval. The radioactivity in each pool represented > 92% of the total radioactivity recovered in urine over the entire collection period.

Technical details of the LC–MS system used in this study are described in Table S2. An LTQ‐Orbitrap‐Elite hybrid mass spectrometer (Thermo Fisher Scientific, Waltham, MA, USA) was used for routine metabolite profiling and accurate mass molecular weight determinations for structural characterization. Samples were analyzed in the full‐scan MS mode for acquisition of the MH+ molecular ion. The high‐resolution, accurate mass capabilities of the instrument allowed metabolite elemental compositions to be determined from the MH+ molecular ion data. Select samples were also analyzed by MS/MS using high‐energy collision dissociation and collision‐induced dissociation for the acquisition of fragmentation data for metabolite structural elucidation. Data‐dependent triggering, based on a list of accurate mass molecular ions of known or suspected metabolites, was used to select precursor masses in a 30‐millimass unit window for subsequent MS/MS scans, allowing fragmentation spectra for multiple metabolites to be acquired in a single run. Electrospray ionization in the positive ion mode was used for all analyses. Full MS ion spectra were collected over the range of 250 to 850 mass‐to‐charge ratio. The acquired data were analyzed using Xcalibur (Version 2.1, Thermo Fisher Scientific).

All radioactivity data were transferred to the Laura software (version 4.0.5.130, LabLogic Ltd., UK) to generate and integrate radiochromatograms. Radioactive peaks for quantitative analysis were manually selected from the plots. The radioactivity in the region encompassing the beginning and ending of the peak was summed. All further calculations were based on radioactivity. The percent of radioactivity (%RA) in a particular peak, *Z*, was calculated as follows:

$$\%\mathrm{RA}\;\text{in}\;Z=\frac{\mathrm{DPM}\;\text{in peak}\;Z}{\text{total}\;\mathrm{DPM}\;\text{in}\;\mathrm{all}\;\text{integrated peaks}}\times 100$$

The concentration or amount of each component was calculated as %RA (expressed as a fraction) multiplied by the total concentration of radioactivity in plasma or the percentage of the dose in the excreta.

#### 2.1.8 PK Analysis

Maximum (peak) observed plasma drug concentration (*C*_max_), time to reach maximum (peak) drug plasma concentration (*T*_max_), terminal plasma elimination half‐life (*T*_1/2_), AUC from time zero to the last measurable concentration sampling time (AUC_last_), AUC from time zero to infinity (AUC_inf_), apparent oral volume of distribution during terminal phase, and apparent clearance from the plasma (CL/F) for plasma binimetinib and *C*_max_, *T*_max_, and AUC_last_ for blood and plasma total radioactivity and plasma AR00426032 (M3) were determined using non‐compartmental PK analysis (Phoenix WinNonlin 6.3, Pharsight, Mountain View, CA, USA).

#### 2.1.9 Safety

Safety assessments consisted of the following: all adverse events (AEs) and serious AEs; hematology, chemistry, and coagulation panels; urinalysis; vital signs; physical examination; cardiac assessments (electrocardiogram [ECG], echocardiogram, or multigated acquisition scan); and ophthalmic assessments (slit lamp examination, visual acuity testing, visual field testing, intraocular pressure, and indirect fundoscopy with dilation) at specified time points due to risk of transient retinopathy with binimetinib administration.

## 3 Results

### 3.1 Demographics and Safety

Six participants were enrolled in the study. All 6 participants (100%) completed the study per protocol. The median age was 30 years (range, 20–34 years). All participants were male, and 4 (66.7%) were white. Mean body mass index was 23.9 kg/m^2^ (range, 21.2–26.8 kg/m^2^). One participant reported clinically relevant ongoing medical conditions, including a history of intermittent mild urticaria (none present at screening or baseline) and vitreous floaters. None of the participants were current smokers or had smoked in the past month.

The single 45‐mg dose of [^14^C]binimetinib administered in this study was generally safe and well tolerated. Three participants (50%) reported ≥ 1 AE during the study. All AEs were grade 1, except presyncope; this was grade 2, considered unrelated to binimetinib treatment and was resolved by the end of the study. Somnolence and anorectal discomfort (both grade 1) were suspected to be related to binimetinib. No deaths, serious AEs, or other significant AEs occurred. No participants discontinued from the study due to an AE. No participants had clinically significant changes in laboratory measurements, vital signs, ECGs, echocardiograms, multigated acquisition scans, or ophthalmology test results.

### 3.2 Mass Balance

**FIGURE 2:**
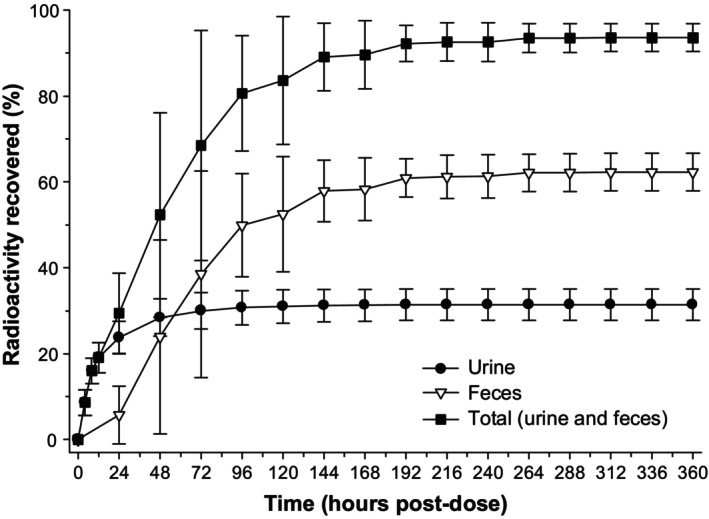
Mean cumulative percent of radioactive dose from all six participants recovered in urine and feces following administration of a single dose of 45 mg of [^14^C]binimetinib to healthy male participants in the pharmacokinetic analysis set (linear scale). Error bars are presented as ± SD and have been staggered.

The mean cumulative excretion of radioactivity from all 6 participants is presented in Figure 2. Overall, good mass balance was achieved in all 6 participants (mean [SD], 93.6 [3.27%]) by day 15. In this study, 31.4% and 62.3% of the radioactivity was recovered in the urine and feces, respectively.

### 3.3 Radiolabeled Blood/Plasma PK

**FIGURE 3:**
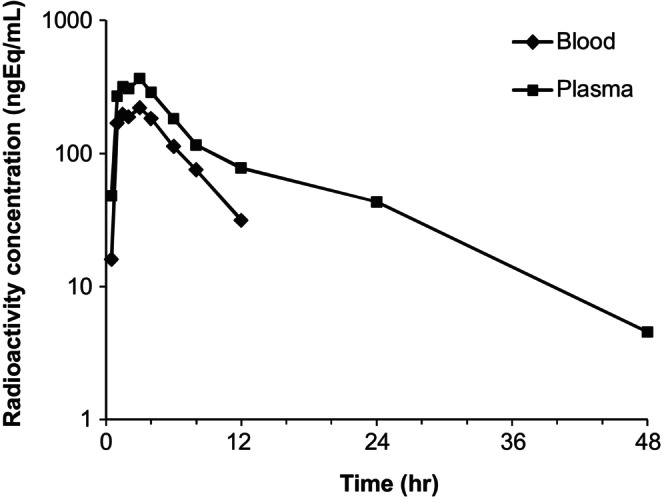
Plasma and whole blood concentration‐versus‐time profiles for a single oral dose of 45 mg (40 μCi) of [^14^C]binimetinib.

The mean concentration‐time profiles of total radioactivity in blood and plasma are shown in Figure 3. The key PK parameters derived from the total radioactivity concentration‐time data in blood and plasma and from binimetinib and AR00426032 concentration‐time data in plasma are summarized in Table 1. For both blood and plasma, the non‐zero concentrations sampled in the terminal phase were insufficient to make reliable estimations of *T*_1/2_ and AUC_inf_ values. The mean blood to plasma concentration ratio for total radioactivity, averaged over a range of post‐dose time points (1–8 h), was 0.63. This value was similar to the mean in vitro distribution of binimetinib, for which a blood to plasma concentration (*C*_blood_/*C*_plasma_) ratio of 0.72 was determined for humans.

**TABLE 1:** Summary statistics of plasma and whole blood PK parameters for total radioactivity.

	Whole blood				Plasma			
	AUC_last_ (ngEq × h/mL)	*C*_max_ (ngEq/mL)	*T*_max_ (h)	*T*_last_ (h)	AUC_last_ (ngEq × h/mL)	*C*_max_ (ngEq/mL)	*T*_max_ (h)	*T*_last_ (h)
*n*	6	6	6	6	6	6	6	6
Mean (SD)	1280 (351)	266 (38.9)	—	—	3060 (498)	425 (53.7)	—	—
Median (Min–max)	1260 (873–1760)	267 (202–318)	2.3 (1–4)	10 (8–12)	3150 (2450–3830)	441 (330–487)	2.3 (1–4)	24 (24–48)

*Note:* Insufficient non‐zero concentrations sampled in terminal phase to estimate *T*_1/2_ and AUCi_nf_ values Calculations were based on the nominal sampling times. Arithmetic mean ± SD for all parameters except: median (range) for *T*_max_, *T*_last_, and *t*_½_. Units for radioactivity parameters are ng Eq/mL (C_max_) or ng Eq × h/mL (AUC).

Abbreviations: AUC, area under the plasma–concentration time profile; AUC_inf_, AUC from time 0 extrapolated to infinite time; AUC_last_, AUC from time zero to the time of the last quantifiable concentration; CL/F, apparent clearance; C_max_, maximum observed plasma concentration; PK, pharmacokinetic; SD, standard deviation; *t*_½_, terminal half‐life; *T*_max_, time for *C*_max_; Vz/F, apparent volume of distribution.

### 3.4 Binimetinib Plasma PK

**FIGURE 4:**
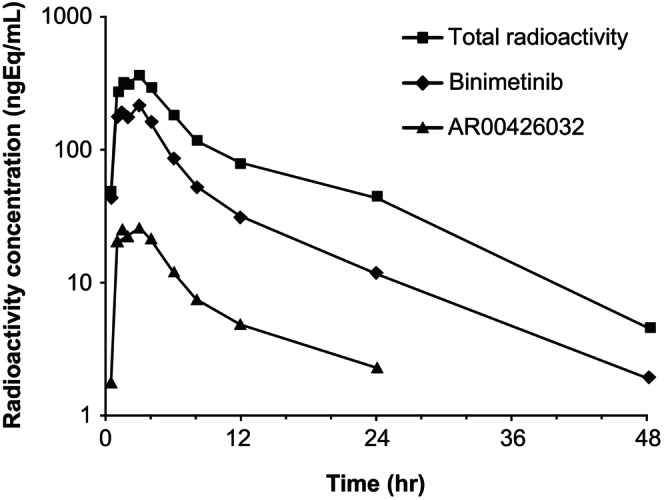
Binimetinib, AR00426032, and radioactivity plasma concentration‐versus‐time profiles for a single oral dose of 45 mg (40 μCi) of [^14^C]binimetinib.

Plasma concentration‐time curves for a single oral dose of 45 mg of radiolabeled binimetinib are presented in Figure 4, alongside both AR00426032 and total radioactivity. Summary statistics for PK parameters of binimetinib are presented in Table 2.

**TABLE 2:** Summary statistics of plasma PK parameters for binimetinib.

	AUC_inf_ (ng × h/mL)	AUC_last_ (ng × h/mL)	AUC_0–24_ (ng × h/mL)	*C*_max_ (ng/mL)	*T*_max_ (h)	*T*_1/2_ (h)	CL/F (L/h)	V_z_/F (L)
*n*	6	6	6	6	6	6	6	6
Mean (SD)	1640 (294)	1620 (290)	1430 (250)	257 (39.5)	—	—	28.2 (4.94)	384 (113)
Median (Min–max)	1630 (1280–2100)	1610 (1270–2070)	1380 (1160–1790)	253 (199–316)	2.3 (1–4)	8.7 (8.1–13.6)	27.7 (21.4–35.0)	374 (252–554)

### 3.5 Metabolite Profiles in Plasma and AR00426032 (M3) Plasma PK

**FIGURE 5:**
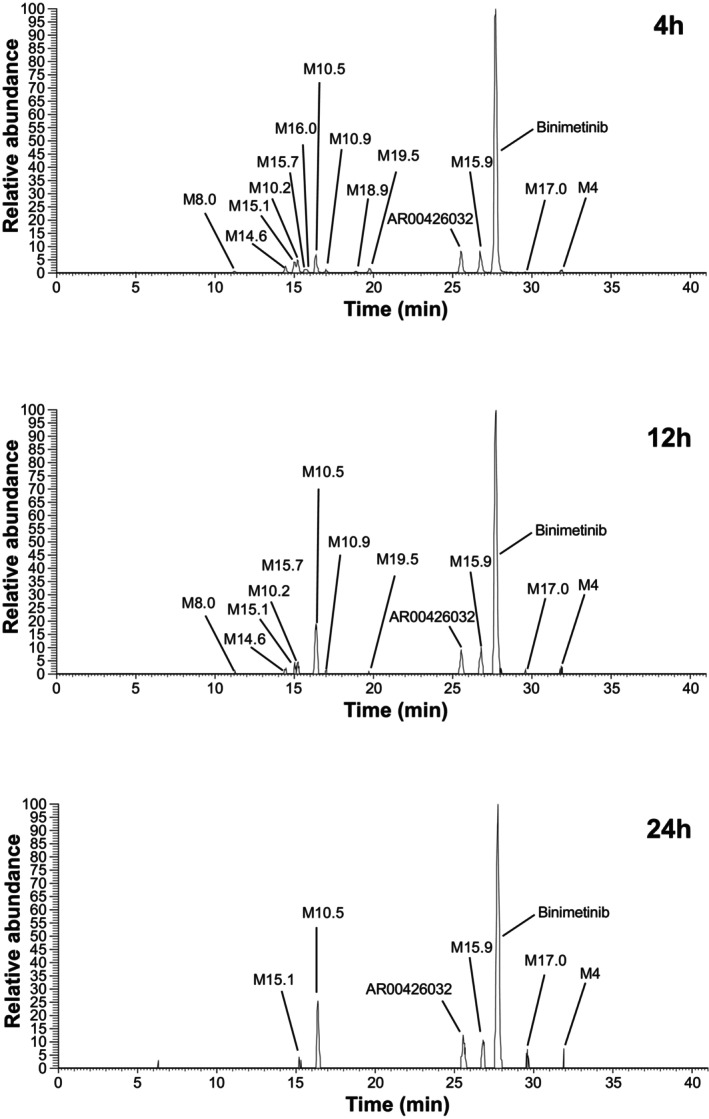
Representative radio‐chromatographic traces of metabolic profile in plasma at 4, 12, and 24 h post‐dose following administration of a single dose of 45 mg (40 μCi) [^14^C]binimetinib to healthy male participants.

After the oral administration of binimetinib to study participants, binimetinib and 14 metabolites were identified in plasma. The primary biotransformation pathways of binimetinib included glucuronidation, N‐dealkylation, amide hydrolysis, and loss of ethane‐diol from the side chain, with secondary pathways including glucuronidation, oxygenation, and dehydrogenation of primary biotransformation products. A representative radio‐chromatographic trace of the metabolite profile in plasma at 4 h post‐dose is shown in Figure 5. AR00426032 (M3), the N‐demethylated form of binimetinib produced by CYP1A2 and CYP2C19, is considered to be an active metabolite, and summary statistics of its PK parameters are presented in Table 3. Due to insufficient non‐zero concentrations sampled in the terminal phase, estimated *T*_1/2_ and AUC_inf_ values were not reported. The amounts of binimetinib and AR00426032 determined by LC–MS/MS had good agreement with the amount determined by LC with radioactivity detection. The ratio of AR00426032 to binimetinib based on mean AUC from time 0–24 h post‐dose values was 0.12 determined by radioactivity and 0.14 determined by MS. This ratio is also in reasonable agreement with what has been observed previously in other studies and clinical trials in patients with cancer (< 23%) [1, 17, 18].

**TABLE 3:** Summary statistics of plasma PK parameters for AR00426032 (M3).

	AUC_last_ (ng × h/mL)	*C*_max_ (ng/mL)	*T*_max_ (h)	*T*_last_ (h)
*n*	6	6	6	6
Mean (SD)	197 (48.3)	34.8 (5.24)	—	—
Median (Min–max)	188 (146–285)	35.3 (26.4–41.8)	1.5 (1–4)	24 (24–24)

The most abundant radiolabeled component was binimetinib, with an average circulating radioactivity in plasma based on an AUC from time 0–24 h post‐dose of 60.2% (53.6%–67.7%). The major circulating metabolites were M10.2 (direct glucuronide of binimetinib; 5.5%), M10.5 (loss of ethane‐diol from side chain, dehydrogenation, and glucuronidation; 6.7%), M3 (AR00426032 [N‐demethylated binimetinib]; 7.3%) and M15.9 (carboxylic acid formed from amide hydrolysis; 7.4%). All other circulating metabolites were present at lower levels, with no individual metabolite contributing > 2% to the total radioactivity AUC (mean values) Table 4. Plasma metabolite profiles based on radioactivity could not be obtained for later time points post‐dose due to limited radioactivity in these samples. However, metabolite profiles based on MS could be obtained for these samples by constructing composite extracted ion chromatograms as shown in Figure 5. Although the peak areas in these chromatograms cannot be used for absolute quantitation, their relative intensities could be used to evaluate changes in concentration over time. Visual inspection of Figure 5 suggests that several metabolites, including M10.5 and M17.0, may have longer half‐lives than binimetinib since their intensities relative to binimetinib increased at later time points (with relative abundance increasing from ≈ 10% to 25% and from ≈ 1% to 5% at 4 and 24 h for M10.5 and M17, respectively).

**TABLE 4:** Plasma AUC values of binimetinib and its metabolites following a single oral dose of 45 mg of [^14^C]binimetinib expressed as ngEq × h/mL (top) and % of total (bottom).

Metabolite/participant	5010‐00006	5010‐00020	5010‐00028	5010‐00034	5010‐00037	5010‐00039	Mean	SD
AUC(0–24 h) ngEq × h/mL								
M8.0	9.70	1.50	41.0	ND	22.8	42.9	19.7	19.1
M14.6	65.2	9.30	54.0	71.2	68.6	42.9	51.9	23.4
M15.1	79.6	19.9	13.9	7.30	7.70	34.8	27.2	27.6
M10.2	128	124	158	148	151	262	162	50.8
M15.7	28.8	33.6	21.5	15.2	22.8	29.9	25.3	6.7
M16.0	26.6	41.3	27.5	43.1	41.9	74.6	42.5	17.4
M10.5	191	193	295	141	139	230	198	58.7
M10.9	ND	16.8	14.5	15.7	23.8	47.5	19.7	15.7
M18.9	ND	10.8	14.8	0.50	11.2	32.9	11.7	12.0
M19.5	24.1	39.8	27.5	36.5	44.1	57.0	38.2	11.9
M24.1	19.4	24.5	54.0	29.1	25.0	84.2	39.4	25.1
M3 (AR00426032)	232	176	238	155	184	322	218	60.5
M15.9	172	158	283	190	199	314	219	63.9
Binimetinib	2120	1630	1800	1530	1420	2050	1760	285
M17.0	14.4	39.8	35.0	29.4	63.4	117.5	49.9	36.8
M4	28.8	65.8	74.5	43.8	57.0	85.7	59.3	20.7
% Radioactivity								
M8.0	0.30	0.10	1.30	ND	0.90	1.10	0.60	0.60
M14.6	2.10	0.40	1.70	2.90	2.80	1.10	1.80	1.00
M15.1	2.50	0.80	0.40	0.30	0.30	0.90	0.90	0.90
M10.2	4.10	4.80	5.00	6.00	6.10	6.80	5.50	1.00
M15.7	0.90	1.30	0.70	0.60	0.90	0.80	0.90	0.20
M16.0	0.90	1.60	0.90	1.80	1.70	2.00	1.50	0.50
M10.5	6.10	7.50	9.30	5.80	5.60	6.00	6.70	1.50
M10.9	ND	0.70	0.50	0.60	1.00	1.20	0.70	0.40
M18.9	ND	0.40	0.50	0.00	0.50	0.90	0.40	0.30
M19.5	0.80	1.50	0.90	1.50	1.80	1.50	1.30	0.40
M24.1	0.60	1.00	1.70	1.20	1.00	2.20	1.30	0.60
M3 (AR00426032)	7.40	6.80	7.50	6.30	7.40	8.40	7.30	0.70
M15.9	5.50	6.10	9.00	7.80	8.00	8.20	7.40	1.30
Binimetinib	67.7	63.1	57.2	62.4	57.2	53.6	60.2	5.10
M17.0	0.50	1.50	1.10	1.20	2.60	3.10	1.70	1.00
M4	0.90	2.60	2.40	1.80	2.30	2.20	2.00	0.60

### 3.6 Metabolite Profiles in Excreta

#### 3.6.1 Feces

An average of 62.3% of the radioactivity was excreted in the feces and included six identified metabolites and parent binimetinib. Binimetinib was the most abundant radioactive component and accounted for a mean of 29.8% (range, 21.1%–45.7%) of the administered radioactive dose. The most abundant metabolites were M4, an ethane‐diol cleavage product, and M15.9, a carboxylic acid formed from amide hydrolysis, accounting for 17.2% and 6.7% of the dose, respectively. All other metabolites were present at ≤ 2.7% of the dose. The chemical structure of metabolite P22.9 could not be determined due to its low abundance in the sample. Further quantitative information on the metabolites is listed in Table S1.

#### 3.6.2 Urine

A total of 14 metabolites and parent binimetinib were identified in urine. Binimetinib was the most abundant radioactive component, accounting for a mean of 6.5% (range, 5.3%–8.1%) of the administered radioactive dose. The mean renal clearance of binimetinib was 1.78 L/h, which was estimated based on the cumulative amount of parent binimetinib eliminated in urine divided by plasma AUC_inf_ of binimetinib. The most abundant metabolites were M10.9 (direct glucuronide of binimetinib), M3 (AR00426032, N‐demethylated binimetinib), and M10.2 (another direct glucuronide of binimetinib), accounting for 6.2%, 5.1% and 4.2% of the dose, respectively. All other metabolites were present at ≤ 3.2% of the dose.

## 4 Discussion and Conclusions

This was an open‐label, single‐center, phase I study to characterize the ADME of binimetinib following a single oral dose of 45 mg containing 40 μCi of [^14^C] in six healthy male participants. This single dose was safe and well tolerated. No significant abnormalities were found in laboratory measurements, vital signs, ECGs, cardiac imaging, or ophthalmology test results.

Mean radioactivity elimination was nearly twice as great in the feces compared with urine. The overall recovery of radioactivity in the excreta of all six participants indicated that good mass balance was achieved [3]. The total percentage of the dose recovered as direct glucuronides of binimetinib (M10.2 and M10.9), formed by UGT1A1, 1A3, 1A9 (primarily M10.9), and 2B7 (primarily M10.2 [data on file]) in the excreta was 11.1%. Mass balance studies of intravenous [^14^C]binimetinib dosing in rats demonstrated the unstable nature of binimetinib's direct glucuronide metabolite M10.9 in the gut (data on file). M10.9 was the most abundant drug‐related compound in the rat bile in bile duct‐cannulated animals, accounting for 90% of the radioactivity recovered. In contrast, M10.9 was only found in trace amounts in the feces of intact rats. Metabolite M4 (amide formed from cleavage of the side‐chain N‐O bond) and unchanged binimetinib accounted for a major portion of the dose recovered in the feces of intact rats but were either absent or present only in trace amounts in the bile. This suggests that direct glucuronide conjugates enter the intestines from the bile but are unstable in the gut and are subsequently converted to binimetinib and eventually M4. A study involving both antibiotic‐treated and control mice indicated that the amide metabolite M4 is most likely due to gut bacteria (consistent with rat ADME data [data on file]). Based on a comparison of the results from these pre‐clinical studies, it is likely that in the current study, much of the dose recovered as binimetinib in the feces (31.7%) was excreted as either M10.2 or M10.9 and then hydrolyzed in the gut to yield binimetinib. Additionally, these studies also suggested the likelihood that in the current study, the dose recovered as metabolite M4 (ethane diol cleavage product) in the feces (18.4%) was excreted as either M10.2 or M10.9 and then hydrolyzed in the gut to yield binimetinib followed by further biotransformation to M4.

The mean percentage of the dose eliminated in the feces as unchanged binimetinib and the M4 amide metabolite (the most abundant metabolite thought to be from bacterially mediated metabolism of binimetinib in the GI tract), normalized to 100% recovery, was 31.7% and 18.4%, respectively. A lower limit on the extent of oral absorption for binimetinib in this study, based on the assumption that the M4 metabolite is formed from binimetinib in the GI tract, can be set at approximately 50% (31.7% plus 18.4%). However, the actual value could be greater if a portion of the unchanged binimetinib in the feces was from absorbed drug that then either was excreted in bile, intestinally secreted, or metabolized to a glucuronide that entered the bile and then was hydrolyzed back to binimetinib in the gut. A simple interpretation of the data is that ≈ 50% of binimetinib is absorbed orally. However, binimetinib behaves like a Biopharmaceutics Classification System Class I drug and exhibits dose proportionality at doses of > 45 mg. Additionally, a formal evaluation of the effect of food on binimetinib PK revealed no clinically meaningful impact, suggesting that binimetinib may be close to fully absorbed at this dose [1, 13]. Therefore, an estimate of the maximum contribution of direct glucuronidation to the clearance of binimetinib in humans is 61.2% (direct glucuronides in the excreta + unchanged binimetinib in the feces + M4 amide metabolite in the feces, that is, 11.1% + 31.7% + 18.4%, respectively), although the actual contribution could be lower.

In urine, 6.5% of the radioactivity was excreted as binimetinib. This resulted in an estimated mean renal clearance of 1.78 L/h, which is 6.3% of the total mean bioavailability‐adjusted CL/F of 28.2 L/h.

In this current study, 17.8% of the dose in the excreta of all metabolites containing the N‐demethylation modification was assumed to have contributed to the clearance of binimetinib by CYP1A2 and CYP2C19. The maximum contribution of direct glucuronidation to the clearance of binimetinib was estimated to be 61.2%. Excretion of unchanged binimetinib into the urine was also estimated to contribute 6.9% to the overall clearance. The remaining 14.2% of the dose in the excreta was associated with metabolites formed from additional modifications of the N‐(2‐hydroxyethoxy) formamide side chain and indirect glucuronidations. The enzymes responsible for these remaining biotransformation reactions have not been determined.

Binimetinib is not thought to undergo enterohepatic (EH) recirculation, despite the formation of direct glucuronide conjugates that are unstable in the GI tract [17]. This is supported by the observation that plasma concentration‐time curves for binimetinib from non‐clinical PK studies are not characteristic of EH cycling (data on file). Furthermore, the M4 metabolite forms in the GI tract prior to reabsorption and therefore counteracts EH cycling. Finally, initial absorption of binimetinib is thought to be rapid, due to its high intrinsic permeability and efflux transporters likely to be saturated due to the high concentration of drug in the GI tract [11, 12, 13]. Parent binimetinib or glucuronide conjugate that reaches the small intestine via the bile will be at a lower concentration, with efflux processes working against EH cycling [11].

Following a single oral dose of [^14^C]binimetinib 45 mg to healthy male participants, the median plasma T_max_ for both radioactivity and binimetinib occurred at 2.3 h. The variability in the plasma exposures of radioactivity and binimetinib between participants both in terms of *C*_max_ and AUC was low (coefficient of variation < 20%). Studies conducted in patients with *NRAS*‐mutant melanoma reported a coefficient of variation of 37.7% and 52.6% for AUC from time zero to tau (dosing interval) and *C*_max_ at steady state, respectively [19]. Although a difference in variability between the healthy volunteer and cancer patient populations can be observed, the controlled nature of a mass balance study regarding strict participant inclusion and exclusion criteria may limit any indication of physiological differences between the two populations that could impact the PK of binimetinib. Differences were also noted between the half‐life reported here (median 8.7 h) and those reported in cancer patients (2.9–4.7 h) [2, 12, 13]. These differences may arise from factors mentioned previously, or because rich terminal phase sampling used in this study to best describe the PK profile was not available in previous clinical studies.

On average, ≈ 60% of the circulating radioactivity AUC in plasma was attributable to parent binimetinib. Fourteen metabolites were found circulating in plasma, with none present at mean levels of > 7.4% of the circulating radioactivity AUC, including the active metabolite M3 (AR00426032). Previous reports suggest that the measurement of circulating metabolite levels relative to total radioactivity is unlikely to raise a safety concern requiring further toxicologic evaluation as these are < 10% of total drug‐related exposure [20].

**FIGURE 6:**
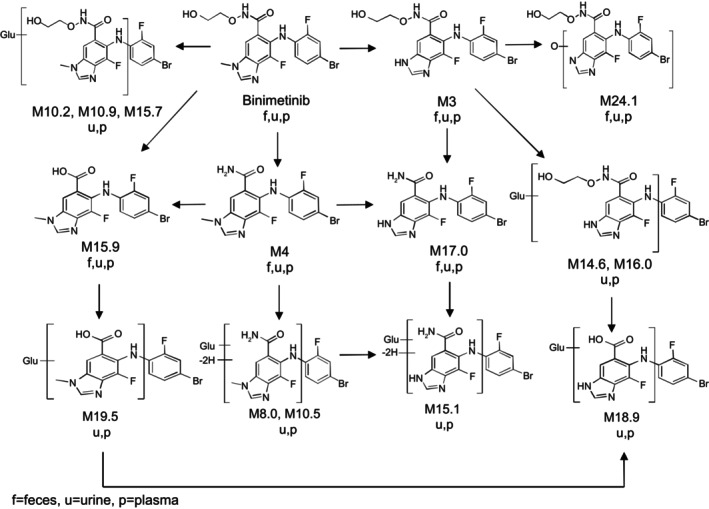
Biotransformation pathways for binimetinib (MEK162).

The primary biotransformation pathways of binimetinib observed in humans were very similar to those observed in preclinical toxicology studies in rats and monkeys (data not shown) and included glucuronidation, N‐dealkylation, amide hydrolysis, and loss of ethane‐diol from the side chain. Secondary biotransformation pathways involving the primary biotransformation products included glucuronidation, oxygenation, and dehydrogenation (Figure 6). Several circulating plasma metabolites previously not observed in rat and monkey ADME studies were identified that circulate at low levels relative to total drug‐related radioactivity (< 2% individually); however, all these metabolites (M14.6, M15.1, M15.7, M16.0, M18.9, M19.5, M24.1), except for M24.1 (mono‐oxygenated derivative of the previously observed M3 metabolite [AR00426032]), were either glucuronides of previously observed metabolites or combinations of previously observed metabolites. Results from this study suggest that the half‐lives of the M10.5 and M17.0 metabolites may be slightly longer than that of binimetinib, but this difference was minor. Overall, the binimetinib metabolites found in this study are unlikely to raise a safety concern requiring further toxicologic evaluation.

In summary, this study based on a single 45‐mg dose of [^14^C]binimetinib exhibited good mass balance recovery and provided clinical data to help characterize the metabolic profile of binimetinib in systemic circulation and excreta. Based on study results, binimetinib is at least ≈ 50% absorbed, but absorption is thought to be significantly higher. Additionally, results of this study supported the design of renal/hepatic impairment studies and indicated that a drug–drug interaction for binimetinib as a victim via UGT1A1 inhibition is possible. The current estimate for the maximum contribution of UGT1A1 to binimetinib metabolism does not account for the involvement of other UGTs in direct glucuronide conjugate formation (that is, this estimate is conservative). This potential interaction was deemed not clinically relevant, as simulations predict a similar *C*_max_ of binimetinib 45 mg in the presence or absence of the UGT1A1 inhibitor atazanavir (400 mg) [12]. Despite the identification of a total of 14 metabolites circulating in plasma, none were present at levels > 10% of the circulating radioactivity AUC, indicating that further toxicological evaluation of metabolites was not required.

## Author Contributions

*Participated in research design*: Renae Chavira. *Performed data analysis*: Dustin Huynh, Erik Hahn, Micaela Reddy, Renae Chavira, Lance Wollenberg. *Wrote or contributed to the writing of the manuscript*: Dustin Huynh, Erik Hahn, Micaela B.Reddy, Renae Chavira, Lance Wollenberg.

## Conflicts of Interest

Dustin Huynh, Erik Hahn, Micaela Reddy, Renae Chavira, and Lance Wollenberg disclose employment with Pfizer. Lance Wollenberg, Micaela Reddy, and Renae Chavira are Pfizer shareholders. Dustin Huynh was contracted by Pfizer to complete this study as part of a Pfizer UCSD Post Doctoral Fellowship.

## Supporting information


**Data S1.** Supporting Information.

## Data Availability

Upon request, and subject to review, Pfizer will provide the data supporting the findings of this study. Subject to certain criteria, conditions and exceptions, Pfizer may also provide access to the related individual de‐identified participant data. See https://www.pfizer.com/science/clinical‐trials/trial‐data‐and‐results for more information.

## References

[1] J. C. Bendell , M. Javle , T. S. Bekaii‐Saab , et al., “A Phase 1 Dose‐Escalation and Expansion Study of Binimetinib (MEK162), a Potent and Selective Oral MEK1/2 Inhibitor,” British Journal of Cancer 116, no. 5 (2017): 575–583, 10.1038/bjc.2017.10. 28152546 PMC5344293

[2] R. J. Sullivan , J. Weber , S. Patel , et al., “A Phase Ib/II Study of the BRAF Inhibitor Encorafenib Plus the MEK Inhibitor Binimetinib in Patients With BRAF(V600E/K)‐Mutant Solid Tumors,” Clinical Cancer Research 26, no. 19 (2020): 5102–5112, 10.1158/1078-0432.Ccr-19-3550. 32669376

[3] US Food and Drug Administration , Clinical Pharmacology Considerations for Human Radiolabeled Mass Balance Studies (Silver Spring, Maryland: Center for Drug Evaluation and Research, 2024), https://www.fda.gov/regulatory‐information/search‐fda‐guidance‐documents/clinical‐pharmacology‐considerations‐human‐radiolabeled‐mass‐balance‐studies.

[4] ClinicalTrials.gov , “A Study to Learn About the Study Medicine Called PF‐07799933 in People With Advanced Solid Tumors With BRAF Alterations (NCT05355701),” 2024, https://clinicaltrials.gov/study/NCT05355701.

[5] ClinicalTrials.gov , “Testing the Use of Fulvestrant and Binimetinib Targeted Treatment for NF1 Mutation in Hormone Receptor‐Positive Metastatic Breast Cancer (A ComboMATCH Treatment Trial) (NCT05554354),” 2024, https://clinicaltrials.gov/study/NCT05554354?cond=breast&intr=binimetinib&rank=6.

[6] ClinicalTrials.gov , “Study of Chemotherapy, With or Without Binimetinib in Advanced Biliary Tract Cancers in 2nd Line Setting (A ComboMATCH Treatment Trial) (NCT05564403),” 2024, https://clinicaltrials.gov/study/NCT05564403?term=binimetinib,%20chemotherapy&page=1&rank=8.

[7] ClinicalTrials.gov , “A Phase 1/2 Study of DCC‐3116 in Patients With RAS/MAPK Pathway Mutant Solid Tumors (NCT04892017),” 2024, https://clinicaltrials.gov/study/NCT04892017.

[8] ClinicalTrials.gov , “JAB‐3312 Based Combination Therapy in Adult Patients With Advanced Solid Tumors (NCT04720976),” 2024, https://clinicaltrials.gov/study/NCT04892017.

[9] ClinicalTrials.gov , “Avelumab With Binimetinib, Sacituzumab Govitecan, or Liposomal Doxorubicin in Treating Stage IV or Unresectable, Recurrent Triple Negative Breast Cancer (InCITe) (NCT03971409),” 2024, https://clinicaltrials.gov/study/NCT03971409.

[10] ClinicalTrials.gov , “Targeted Therapy Directed by Genetic Testing in Treating Patients With Locally Advanced or Advanced Solid Tumors, The ComboMATCH Screening Trial (NCT05564377),” 2024, https://clinicaltrials.gov/study/NCT05564377.

[11] M. S. Roberts , B. M. Magnusson , F. J. Burczynski , and M. Weiss , “Enterohepatic Circulation: Physiological, Pharmacokinetic and Clinical Implications,” Clinical Pharmacokinetics 41, no. 10 (2002): 751–790, 10.2165/00003088-200241100-00005. 12162761

[12] US Food and Drug Administration , Highlights of Prescribing Information: MEKTOVI (Binimetinib) Tablets (Boulder, CO: FDA, 2018), https://www.accessdata.fda.gov/drugsatfda_docs/label/2018/210498lbl.pdf.

[13] K. Watanabe , S. Otsu , Y. Hirashima , et al., “A Phase I Study of Binimetinib (MEK162) in Japanese Patients With Advanced Solid Tumors,” Cancer Chemotherapy and Pharmacology 77, no. 6 (2016): 1157–1164, 10.1007/s00280-016-3019-5. 27071922

[14] Y. Gan , J. P. A. Baak , T. Chen , et al., “Supersaturation and Precipitation Applicated in Drug Delivery Systems: Development Strategies and Evaluation Approaches,” Molecules 28, no. 5 (2023): 2212, 10.3390/molecules28052212. 36903470 PMC10005129

[15] Pfizer , “Data on File,” 2024.

[16] R. A. Hamilton , W. R. Garnett , and B. J. Kline , “Determination of Mean Valproic Acid Serum Level by Assay of a Single Pooled Sample,” Clinical Pharmacology and Therapeutics 29, no. 3 (1981): 408–413, 10.1038/clpt.1981.56. 6781809

[17] M. B. Reddy , M. B. Bolger , G. Fraczkiewicz , et al., “PBPK Modeling as a Tool for Predicting and Understanding Intestinal Metabolism of Uridine 5′‐Diphospho‐Glucuronosyltransferase Substrates,” Pharmaceutics 13, no. 9 (2021): 1325, 10.3390/pharmaceutics13091325. 34575401 PMC8468656

[18] J. Piscitelli , M. B. Reddy , L. Wollenberg , et al., “Evaluation of the Effect of Modafinil on the Pharmacokinetics of Encorafenib and Binimetinib in Patients With BRAF V600‐Mutant Advanced Solid Tumors,” Cancer Chemotherapy and Pharmacology 94, no. 3 (2024): 337–347, 10.1007/s00280-024-04676-2. 38878209 PMC11420244

[19] M. Schuler , L. Zimmer , K. B. Kim , et al., “Phase Ib/II Trial of Ribociclib in Combination With Binimetinib in Patients With NRAS‐Mutant Melanoma,” Clinical Cancer Research 28, no. 14 (2022): 3002–3010, 10.1158/1078-0432.Ccr-21-3872. 35294522 PMC9365377

[20] US Food and Drug Administration , Safety Testing of Drug Metabolites (Silver Spring, Maryland: Center for Drug Evaluation and Research, 2020), https://www.fda.gov/regulatory‐information/search‐fda‐guidance‐documents/safety‐testing‐drug‐metabolites.

