# Supplementary material for: The Absorption, Distribution, Metabolism, and Excretion of Binimetinib Following a Single Oral Dose of [ 14C]Binimetinib 45 mg in Healthy Male Participants
Source: Pharmacol Res Perspect. 2025 Jan 30;13(1):e70061. doi: 10.1002/prp2.70061 (PMC11780716; doi:10.1002/prp2.70061)
Supplement: Supplementary file 1 — Data S1. Supporting Information. [file PRP2-13-e70061-s001.docx]

**Supplementary Information**

**Supplementary Table S1.** Sample collection scheduling

| PK collection number | **Blood sample collection** | | **Urine and feces collection** | |
| --- | --- | --- | --- | --- |
|  | **Tube 1:** total radioactivity, whole blood (2 mL/sample)  **Tube 2:** total radioactivity, plasma (3 mL/sample)  **Tube 3:** PK analysis, plasma (2 mL/sample)  **Tube 4:** metabolite characterization, plasma (10 mL/sample) | | Total radioactivity and metabolite characterization | |
|  | **Day** | **Time point post-dose (hours)** | **Day** | **Time period post-dose (hours)** |
| 1 | -1 | Pre-dose | -1 | Pre-dose |
| 1 | 1 | 0.5 | 1 | 0–4 |
| 1 | 1 | 1 | 1 | 4–8 |
| 1 | 1 | 1.5 | 1 | 8–12 |
| 1 | 1 | 2 | 1–2 | 12–24 |
| 1 | 1 | 3 | 2–3 | 24–48 |
| 1 | 1 | 4 | 3–4 | 48–72 |
| 1 | 1 | 6 | 4–5 | 72–96 |
| 1 | 1 | 8 | 5–6 | 96–120 |
| 1 | 1 | 12 | 6–7 | 120–144 |
| 1 | 2 | 24 | 7–8 | 144–168 |
| 1 | 3 | 48 | 8–9 | 168–192 |
| 1 | 4 | 72 | 9–10 | 192–216 |
| 1 | 5 | 96 | 10–11 | 216–240 |
| 1 | 6 | 120 | 11–12 | 240–264 |
| 1 | 7 | 144 | 12–13 | 264–288 |
| 1 | 8 | 168 | 13–14 | 288–312 |
| 1 | 9 | 192 | 14–15 | 312–336 |
| 1 | 10 | 216 | 15–16 | 336–360 |
| 1 | 11 | 240 | 16–17 | 360–384 |
| 1 | 12 | 264 | 17–18 | 384–408 |
| 1 | 13 | 288 | 18–19 | 408–432 |
| 1 | 14 | 312 | 19–20 | 432–456 |

Direct venipuncture in the forearm was used to collect blood samples into 4 separate potassium-EDTA–containing tubes for metabolite characterization, total radioactivity measurement, and parent drug analysis.

Urine samples were collected in labeled polypropylene containers.

All bowel movements were collected in pre-weighed, labeled polypropylene containers up to 456 hours after drug administration. The total weight of each fecal sample at each interval (e.g., pre-dose, 0 to 24 hours, and 24 to 48 hours) was recorded. Any additional soiled toilet paper was collected and stored separately in a plastic bag. All plasma samples, urine aliquots, and homogenized feces aliquots were stored at or below −20°C ±10°C.

**Supplementary Table S2.** LC-MS/MS technical details

| **Analytical column:** Phenomenex Synergi Hydro-RP, 80Å, 4.6 x 150 mm, 4 μM (Phenomenex Inc., Torrance, CA)  **Pre-column:** Phenomenex SecurityGuard C-18, 4 x 3 mm  **Column temperature:** 40°C  **Flow rate:** 1 mL/min  **Gradient elution programme:** 0 to 1 minute, 1% B; 1 to 3 minutes, 1% to 14% B; 3 to 25 minutes, 14% to 30% B; 25 to 32 minutes, 30% to 40% B; 32.1 to 35 minutes, 95% B; 35.1 to 41 minutes, 1% B | | |
| --- | --- | --- |
| Time (minutes) | Mobile phases | |
|  | **Solvent A:** 10 mM ammonium acetate, 0.1% acetic acid, pH 4.5 (%) | **Solvent B:** Acetonitrile (%) |
| Initial | 99 | 1 |
| 1 | 99 | 1 |
| 3 | 86 | 14 |
| 25 | 70 | 30 |
| 32 | 60 | 40 |
| 32.1 | 5 | 95 |
| 35 | 5 | 95 |
| 35.1 | 99 | 1 |
| 41 | 99 | 1 |

Reconstituted plasma and fecal extracts and processed urine samples were sonicated and vortexed then analyzed by a Waters Acquity ultra-performance LC system (Waters Corp., Milford, MA, USA) coupled to an LTQ-Elite hybrid mass spectrometer (ThermoFisher Scientific Corporation, Waltham, MA, USA) and a Leap Collect PAL fraction collector (Leap Industries, Carrboro, NC, USA).

Plasma extracts were suspended in 4.4 mL of distilled water and injected through a manual injector equipped with a 5-mL sample loop (Rheodyne model 7725, Waters Corp., Milford, MA, USA). The entire content of the loop was introduced onto a trapping column (YMC-Pack DSO-AQ, 50 × 4.6 mm, 3 µ, 12 nm, YMC Co. Ltd., Allentown, PA, USA) using a Shimadzu LC-20AD pump at a flow rate of 0.6 mL/min and an isocratic mobile phase consisting of 3% acetonitrile in water. After trapping the sample for 10 minutes at the head of the column, the trapping column was back flushed onto the analytical column for separation.
